# A Holistic Framework for Evaluating Food Loss and Waste Due to Marketing Standards across the Entire Food Supply Chain

**DOI:** 10.3390/foods13203273

**Published:** 2024-10-15

**Authors:** Evripidis P. Kechagias, Sotiris P. Gayialis, Nikolaos Panayiotou, Georgios A. Papadopoulos

**Affiliations:** Sector of Industrial Management and Operational Research, School of Mechanical Engineering, National Technical University of Athens, 15780 Athens, Greece; sotga@mail.ntua.gr (S.P.G.); panayiot@central.ntua.gr (N.P.); gpapado@mail.ntua.gr (G.A.P.)

**Keywords:** food waste, food loss, marketing standards, supply chain management, food systems, sustainable food chain

## Abstract

This paper addresses the critical and urgent need to reduce food losses and waste (FLW) resulting from stringent marketing standards. It proposes a comprehensive and actionable framework grounded in the three pillars of sustainability—environmental, economic, and social—to effectively evaluate FLW across the entire food supply chain. The paper involves a thorough review of existing marketing standards, including research on FLW due to marketing standards, and proposes the implementation of targeted key actions within four key food sectors: fruits, vegetables, dairy, and cereals. The study provides a detailed analysis of the significant impact marketing standards have on FLW at various stages of the supply chain, including primary production, processing, retail, and consumption. By focusing on these critical points, the research underscores the necessity of addressing marketing standards to achieve meaningful reductions in FLW. The proposed framework aims to foster improved business practices and drive the development of innovative, sector-specific solutions that balance sustainability goals with economic viability. The holistic approach followed for this research lays the foundation for ensuring that the proposed framework is adaptable and practical, leading to measurable improvements in reducing FLW and promoting sustainability across the food industry.

## 1. Introduction

Food waste is a pervasive and multifaceted challenge that permeates every stage of the food supply chain, exerting detrimental effects on the sustainability of the food industry in environmental, economic, and social dimensions [1]. The Food and Agriculture Organization of the United Nations (FAO) characterizes food waste as predominantly stemming from consumption inefficiencies, encompassing factors such as overproduction, expiration dates, and shifting consumer preferences [2]. This wasteful phenomenon manifests at various points in the food supply chain, spanning primary production, retail, and food service [3]. Conversely, food losses result from inefficiencies during production and processing, such as spoilage during transportation, damage during harvesting, or inadequate storage facilities [4]. These losses occur before the food even reaches consumers, underscoring the complexity of the challenge [5]. Recognizing the diverse causes of food waste and losses opens the door to targeted strategies and solutions that address each issue specifically. Such solutions may involve enhancing consumer education and behavior, optimizing supply chain logistics, investing in advanced storage and processing facilities, and implementing sustainable agricultural practices [6].

Reducing food waste and losses emerges as a pivotal step in fostering a more sustainable and equitable global food system [7]. However, pervasive issues like a lack of awareness about the magnitude of the problem and ingrained consumer behaviors, particularly in developed countries where food waste is accepted as a societal norm [8,9], present significant barriers to progress [10]. FAO estimates indicate that a staggering 1.3 billion tons of food, equivalent to one-third of all food produced for human consumption, is lost or wasted at each stage of the food supply chain [11]. Food loss and waste occurs in all stages of the food supply chain, and according to Eurostat’s latest estimations [12], quantification of European food waste levels reveals that 69% of EU food waste arises in the household (53%), food service (9%), and retail sectors (7%), with production (11%) and processing sectors (20%) contributing the remaining 31%.

Among the various factors influencing food waste, marketing standards play a crucial role in shaping consumer choices and perceptions. These standards, which regulate protected terms and labels, are designed to furnish consumers with information while striving to establish a standardized and satisfactory quality for agri-food products in the market [13]. Available in diverse forms, including international, European, national, and private standards, marketing standards sometimes clash with food waste reduction objectives. It is imperative to acknowledge and assess the trade-offs between the goals of marketing standards, primarily focused on ensuring high-quality food provision, and the imperative to minimize food waste. The tension arises from the possibility that stringent standards may contribute to discarding edible food deemed imperfect but still safe for consumption [14]. In this context, consumer behavior emerges as a significant factor influencing food waste. Marketing solutions, encompassing the introduction of new standards and innovative sales channels, hold the potential to mitigate food waste by providing enhanced market access for foods that do not meet conventional marketing standards but remain safe for consumption. The dynamic interplay between marketing standards and food waste reduction underscores the need for a nuanced understanding of the mechanisms influencing both realms. Marketing standards, which act as a regulatory framework for product quality and information dissemination, are critical for shaping consumer choices and fostering confidence in the agri-food market. These standards, however, sometimes inadvertently contribute to the discarding of food items that fall outside prescribed aesthetic or quality norms [15].

The conflict between food waste reduction and marketing standards becomes particularly evident when considering cosmetic imperfections or deviations from standardized appearances. Fruits and vegetables, for instance, may be discarded due to blemishes, irregular shapes, or sizes that do not conform to traditional expectations, despite being perfectly safe and nutritious [16]. This discord highlights the potential tension between the pursuit of marketable aesthetics and the imperative to minimize food waste. Addressing this challenge requires a comprehensive assessment of existing marketing standards and their implications for food waste. It is essential to explore innovative solutions that align consumer expectations with sustainability goals [17]. One avenue involves revisiting and redefining aesthetic criteria within marketing standards to encompass a broader spectrum of visual imperfections deemed acceptable by consumers. Consumer education campaigns can play a pivotal role in shifting societal norms, encouraging the acceptance of “imperfect” but perfectly edible produce [18]. Furthermore, advancements in technology offer tools to streamline supply chain processes, reduce losses, and enhance the marketability of food items that would otherwise be discarded. Precision agriculture, data analytics, and blockchain technologies enable better inventory management, real-time tracking, and traceability, thereby reducing the likelihood of overproduction and enhancing overall supply chain efficiency [19].

Private marketing standards, often developed and owned by non-governmental entities, present a unique set of challenges and opportunities. These standards may conflict with government regulations, varying in both stringency and scope. However, they can also serve as instruments for ensuring sustainable food supply chains with minimized food waste [20]. Voluntary sustainability standards (VSS) exemplify private standards that could play a pivotal role in reducing food waste. These standards are designed to ensure that products are produced, processed, or transported sustainably, contributing to specific environmental, social, and economic targets [21].

Recent research efforts have increasingly focused on analyzing the impact of marketing standards on food FWL, emphasizing policy interventions, technological innovations, and shifts in consumer behavior. In their policy analysis of food waste management from around the world, Shen et al., (2024) [22] highlight the current gaps to include the call for enhanced legislation covering food waste in various countries, especially developing ones such as China. This, they argue, requires policies at the national level that will ensure sustainable development. Mokrane et al., (2023) [23], for instance, build on social network analysis and bibliometrics to chart the expansion of scholarly production on FWL. Their study emphasizes moving from the traditional downstream type of prevention to new and improved modern better upstream type of prevention that is more environmentally sustainable, such as the circular economy approach, hence signaling the need for interdisciplinarity. Pérez-Marroquín et al., (2023) [24] proves the asymptomatic ambulatory travel potential of functional beverages made from agro-food waste, thereby showing that food waste could be utilized effectively in creating new products. Also, Sanders (2023) [25] studies dynamic pricing as a retail approach to minimizing food waste and concludes that it yields much better outcomes than organic waste landfill bans not only in terms of waste reduction but also when it comes to increasing profit margins. Dsouza et al., (2023) [26] deal with food safety, reputation, and regulation and show that private incentives as well as mandatory release of information regarding food safety enhance consumer protection and trust. Finally, Facchini et al., (2023) [27] presented research on interventions for minimizing food waste and insist that there is a need to solve the problem through policy and technology and get rid of complacency involving both food security and food waste management. These studies collectively demonstrate the multi-dimensional approach that is necessary for addressing FWL, including regulation, technology, and changes in market and consumer behavior.

Delving into the complexities of the food waste challenge, this paper aims to provide a framework for evaluating marketing standards and their impact on food waste reduction, offering insights into potential solutions and areas for future research. This paper also investigates the intricacies of the relationship between marketing standards and food waste reduction, offering insights into potential modifications, innovations, and collaborative approaches. By providing a holistic framework, the paper aims to contribute to the ongoing discourse on developing sustainable and equitable food systems that address the global challenge of food waste while meeting the diverse needs of consumers and industry stakeholders. The paper provides a significant contribution in identifying a clear and practical roadmap to consider the three aspects of sustainability, namely, the environmental, economic, and social impacts, and creating a framework for measuring the FLW caused by strict marketing standards throughout the food value chain. It builds on previous work by providing more development on the unsuccessful integration of contemporary marketing conventions and FLW by highlighting the aesthetics-fueled discarding of cosmetically appealing, inoffensive food. This paper not only explains the negative impacts of these standards but also brings practical strategies for an amendment of less aesthetic criteria and technological advances, like artificial intelligence and blockchain, to make and improve the traceability system to decrease waste. Also, it focuses on the roles of consumers and other stakeholders in countering the FLW problem and presents stakeholder- and consumer-tailored multi-sector interventional strategies in four major food sectors, including fruits and vegetables, dairy products, and cereals, and makes a valuable contribution to discussions on how to build sustainable food systems.

## 2. Current Relationship of Food Waste and Marketing Standards and Solutions for Improvement

### 2.1. Impact of Marketing Standards on Food Losses and Waste

Marketing standards have been identified as one of the main drivers of food losses and waste in the EU, as they often impose strict requirements on the size, shape, labels, packaging, and appearance of foods, leading to perfectly edible products being discarded. A better understanding of the extent to which marketing standards contribute to food losses and waste constitutes an imperative need, as well as identifying the specific types of products and markets that are most affected. This involves analyzing data on food waste and losses throughout the supply chain, from primary production to consumption. By gaining a better understanding of the impact of marketing standards on food losses and waste, policymakers and stakeholders can develop targeted interventions to reduce losses and waste and improve the sustainability of the food system. According to the environmental organization, Feedback EU, global food loss and waste equaled 8–10% of global GHG emissions and cost the world about USD 1 trillion per year [28]. FAO estimates that 1.3 billion tons of food, or one-third of all food produced for human consumption, is lost or wasted at each stage of the food supply chain. Global quantitative food losses and waste per year, according to FAO, are roughly 45% for roots and tubers, fruits and vegetables, 35% for fish and seafood, 30% for cereals, 20% for oil seeds and pulses, meat, and dairy products [11].

This is a significant amount of food waste, which not only has economic and environmental consequences but also contributes to food insecurity and hunger. Food waste occurs in all stages of the food supply chain, and according to Eurostat’s latest estimations [12], quantification of European food waste levels reveals that 71% of EU food waste arises in the household, food service, and retail sectors, with production and processing sectors contributing the remaining 29%. The impact of marketing standards on food losses and waste is a complex issue, and there have been various efforts to examine and understand it. One example is the partnership of Carrefour with Too Good to Go, started in 2020, in several European countries to sell boxes of unsold, surplus food at a discounted price. The initiative allowed Carrefour to reduce food waste by selling surplus products that would have otherwise been thrown away due to marketing standards. At the same time, it provided customers with a more affordable way to purchase food, reducing their own food waste and increasing their access to healthy and sustainable options. In Italy, this coloration has managed to sell over 278,000 of such boxes, only in 2022 [29].

Another example is the EU-funded research project REFRESH, which ran from 2015 to 2019 and aimed to develop and implement strategies to reduce food waste across the entire supply chain. The project involved collaboration between researchers, businesses, and governments across 12 European countries and resulted in the development of tools and resources to help businesses and policymakers tackle food waste [30]. However, there are still gaps in current research efforts. One major gap is the lack of comprehensive data on food losses and waste at various stages of the supply chain. EU food waste statistics have challenges to represent the recent years’ data and focus on measuring the environmental impact of food waste (wasted volume).

However, to tackle the problem effectively, it is also required to measure and analyze the economic impact of food losses and waste. This makes it difficult to accurately measure the impact of marketing standards on food losses and waste in these regions. Additionally, there is a need for more research into the social and behavioral factors that contribute to food losses and waste, such as consumer attitudes and preferences, as well as the impact of policy interventions on reducing food losses and waste. There is also limited research on the potential of new technologies and innovations to reduce food losses and waste caused by marketing standards. Finally, there is a clear lack of collaboration and coordination among stakeholders to address the complex and interconnected factors that contribute to food losses and waste related to marketing standards.

### 2.2. Underlying Reasons for Setting-Up Private Marketing Standards and Impact on Food Waste Generation

Private marketing standards are often established by businesses to differentiate their products from competitors’ products and to appeal to consumers who may have specific preferences for certain characteristics, such as size, shape, color, or texture. These standards are voluntary and are not legally binding, but they may be adopted by industry associations or other groups to promote consistency across the market [31]. One of the main reasons for setting up private marketing standards is to improve the quality and safety of products for consumers. According to several studies, private standards aid in enhancing traceability, minimizing product defects, identifying and managing food safety concerns at an early stage during production, and decreasing the likelihood of consignment refusal [32,33]. All these factors have the potential to contribute to the reduction of food losses and waste.

The market also includes minimum quality standards (MQS), which are established by governments and may also play a role in ensuring that products meet certain safety and quality requirements [34]. However, private marketing standards can go beyond minimum standards and may include additional criteria that are important to consumers, such as sustainability or ethical sourcing. This way, private marketing standards can also contribute to food losses and waste. For example, standards that require uniformity in appearance may lead to the rejection of fruits and vegetables that are perfectly edible but do not meet the desired shape or size [35]. This can result in significant amounts of food being discarded at the farm level or rejected by retailers, contributing to food waste [36]. Therefore, as it becomes clear, further research is needed to understand the role and significance of private standards and their impact towards generating food losses and waste. There can also be conflicts between private marketing standards and minimum quality standards that can impact welfare and competition, particularly in developing countries. MQS can reduce welfare if consumers are able to detect quality at the time of purchase and if MQS increases the market power of firms producing differentiated products.

Developing countries may also be at a disadvantage if they are less able to compete and if it is costly for them to implement marketing standards [37]. In some cases, more stringent marketing standards may lead to higher concentration among exporters, and smaller firms/farms may not be able to comply with standards [38]. On the other hand, the more incentives increase for firms to set up private standards, the lower the level of MQS. This is because the gains from product differentiation are inversely related to the stringency of MQS. In the absence of public MQS, firms will have incentives to introduce private standards as the gains are greatest. However, increased stringency of MQS may reduce the gains from private standards and lessen their use [39]. The role of private marketing standards can also affect consumer expectations. If consumers are accustomed to seeing only perfectly uniform fruits and vegetables in stores, they may perceive irregularly shaped or sized produce as inferior or less desirable. This can lead to a preference for uniformity and contribute to the rejection of perfectly edible but visually imperfect produce, increasing food losses and waste [40]. Overall, the impact of private standards on food losses and waste is complex and depends on various factors, including the specific standards in place, the level of enforcement, and the behavior of stakeholders in the supply chain. It is important, therefore, to better understand the underlying reasons for setting private standards, consider the trade-offs between private and public standards, and evaluate their impact on food losses and waste.

### 2.3. Solutions for Improving the Business Potential of Safe-to-Eat Foods That Do Not Meet Marketing Standards

Safe-to-eat foods that do not meet marketing standards are food products that do not meet the aesthetic or size requirements set by marketing standards but are still safe and edible for human consumption. These may include, for example, fruits and vegetables with slight blemishes, odd shapes, or discolorations, and meat or dairy products with minor imperfections in texture or color. Despite being perfectly safe and nutritious, these foods are often discarded by producers, retailers, and consumers due to their appearance, resulting in significant food losses and waste [41]. However, there is a growing awareness of the environmental and social impact of food losses and waste, and efforts are being made to find solutions to reduce losses and waste and make better use of safe-to-eat foods that do not meet marketing standards. There are several potential solutions for improving the business potential of safe-to-eat foods that do not meet marketing standards. One solution is to sell these products through alternative channels, such as farmers’ markets [42], community-supported agriculture programs [43], or online platforms [44]. By selling directly to consumers, farmers and producers can avoid the restrictions of marketing standards and offer products that may have cosmetic imperfections but are otherwise safe to eat.

Another solution is to process these products into value-added products such as jams, sauces, smoothies, or pickles. This can increase the shelf life of these products and allow farmers and producers to sell them at a higher price point. Collaboration between farmers and food businesses is also a potential solution [42]. For example, farmers could work with food processing companies or restaurants to transform their imperfect produce into food products or meals. This could benefit both parties, as farmers would be able to sell their produce that would otherwise be rejected due to marketing standards, while businesses could offer more sustainable and affordable options to their customers. Donating safe-to-eat foods that do not meet marketing standards to charitable organizations is another solution [45]. This not only reduces food losses and waste but also helps to feed people who may not have access to nutritious food. Another important factor concerns educating and raising awareness among consumers about the safety and nutritional value of foods that do not meet marketing standards, which can help reduce the stigma associated with such products [46]. This can encourage consumers to purchase and consume these products, reducing food waste and improving food security. Additionally, policy changes such as revising marketing standards to allow for more variation in size, shape, and appearance of produce can also help reduce food losses and waste. This could allow more food to be sold and consumed while still meeting basic quality and safety standards.

At the same time, incentives such as tax credits or subsidies can be offered to companies that reduce food losses and waste by utilizing safe-to-eat foods that do not meet marketing standards. Finally, there is also potential for technological solutions to reduce food losses and waste [47]. One such solution is smart packaging, which can include sensors that monitor the freshness and quality of the food. This allows for more accurate labeling and reduces the likelihood of food losses and waste due to premature disposal. Another solution is artificial intelligence (AI) labeling, which uses artificial intelligence to assess the quality of produce, including its color, texture, and overall appearance. This technology can identify products that do not meet marketing standards but are still safe to eat, allowing for them to be sold at a discounted price or processed into other food products. Advanced sorting and processing technologies can also identify and separate safe-to-eat foods from those that do not meet marketing standards. This can include optical sorting machines that can detect blemishes or discoloration and processing techniques such as freezing, canning, or juicing. By utilizing these technological solutions, businesses can reduce food losses and waste and increase their profitability by finding new markets for safe-to-eat foods that do not meet marketing standards.

### 2.4. Sustainability Awareness, Engagement, and Behavioral Change

The sustainability of food systems is a complex issue that depends on several factors, including resource efficiency, conservation of natural resources, rural development, social wellbeing, ecosystem resilience, and responsible governance. According to the Institute of Innovation and Technology (EIT) 2021 report [48], only 47% of Europeans express confidence in the integrity of food products. Despite 76% of Europeans expressing motivation to live sustainably, only 51% consider sustainability when making food choices. This highlights an “attitude-behaviour gap” where consumers may desire to make sustainable choices but struggle to make impactful lifestyle changes. Additionally, while 37% of Europeans are open to adopting new foods, the majority remain hesitant, indicating the need for the food system to promote trust in food innovation [48].

As it becomes clear, there is a great need for sustainability awareness, engagement, and behavioral change. Awareness is essential to ensure that consumers and food supply chain actors understand the importance of reducing food losses and waste and the role of marketing standards in this process. Engagement is necessary to get consumers and actors involved in the process of reducing food losses and waste and to encourage them to take action and make changes [49]. Behavioral change is crucial to ensure that consumers and actors implement the changes they have learned and make them a permanent part of their daily routine. Consumers need to be educated on the impact of marketing standards on food losses and waste and how they can reduce waste by accepting safe-to-eat foods that do not meet the cosmetic standards. They can also make conscious choices to buy and consume more locally sourced and in-season produce, reducing the need for cosmetic standards and improving the overall sustainability of food systems [50].

Additionally, engaging with consumers through campaigns, social media, and other communication channels can raise awareness and encourage behavioral changes towards responsible food consumption [51]. Food supply chain actors can also play a significant role in reducing food losses and waste due to marketing standards by adopting sustainable practices that prioritize the reduction of losses and waste [52]. For example, food manufacturers can use advanced sorting and processing methods to identify and utilize safe-to-eat produce that does not meet cosmetic standards, while retailers can offer discounts on these products to incentivize consumers to buy them. Implementing a more circular economic approach, such as composting or repurposing unsold products can also reduce food losses and waste and improve sustainability.

## 3. Framework for Reducing and Preventing Food Waste Based on Evaluating the Marketing Standards

The European Union has set a target to reduce food waste by 50% by 2030, in line with the United Nations’ Sustainable Development Goals [53]. The EU’s Farm to Fork strategy, which was launched in 2020, also aims to create a more sustainable food system by reducing food waste and promoting sustainable agriculture practices. In this context, this research proposes a concrete framework across the whole food chain in order to evaluate food losses and waste due to marketing standards, taking into account social, economic, and sustainability factors.

### 3.1. Framework Analysis

**Figure 1:**
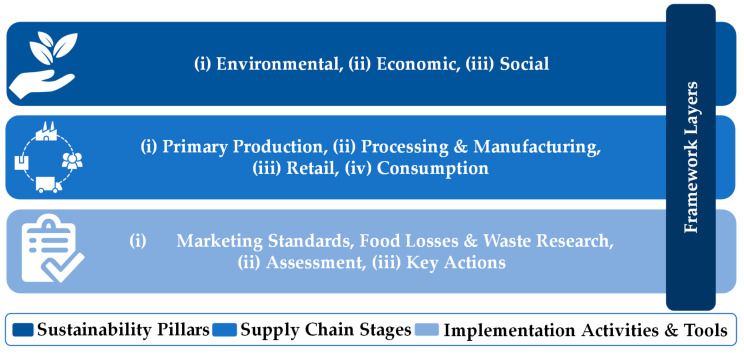
The layers of the framework.

The framework consists of three main layers, as seen in Figure 1: the sustainability pillars, the supply chain stages, and the activities and tools required for effective implementation. Reducing food losses and waste related to marketing standards is an important issue for the framework for several reasons. Firstly, it can help to reduce the environmental impact of food production. When food is wasted, all the resources used to produce it, such as land, water, and energy, are also wasted. By reducing food losses and waste, we can reduce the overall environmental impact of food production and promote a more sustainable food system. Secondly, reducing food losses and waste can also have economic benefits. It also represents a loss of value for producers and retailers, and by reducing losses and waste, we can increase the efficiency of the food system and help to reduce costs for both producers and consumers. This can also contribute to job creation and economic growth, particularly in the agriculture and food processing sectors. Finally, reducing food losses and waste can have social benefits as well. Food losses and waste represent a significant missed opportunity to address food insecurity and poverty. By reducing losses and waste, we can ensure that more food is available for those in need and help to promote food security and social welfare. Therefore, the framework has the potential to contribute to a more sustainable, efficient, and equitable food system.

The first layer of the framework concerns the research aim to tackle the issue of food losses and waste related to marketing standards in a sustainable manner by addressing all three sustainability pillars: (1) environmental, (2) economic, and (3) social. The first pillar aims to minimize the environmental impact of food losses and waste, such as the carbon footprint caused by food that is produced and discarded. The framework focuses on exploring ways to reduce the environmental impact of food losses and waste by integrating innovative solutions that promote circular economy principles. The second pillar focuses on the economic impact of the food supply chain activities, including the long-term viability of food systems. It will help businesses reduce their costs associated with food losses and waste, such as disposal fees, lost revenue, and wasted resources. The framework proposes exploring solutions that are economically sustainable, such as identifying opportunities for businesses to repurpose food waste as a resource or to optimize production processes to reduce waste. The framework also considers how changes in marketing standards can impact the economics of food production and distribution, including the cost of packaging, transportation, and storage. The third pillar is concerned with the impact of human activities on society, including social justice and equity. The framework proposes actions to improve access to food and reduce hunger in order to reduce food poverty. By reducing food losses and waste, more food can be made available to those who really need it. Additionally, the framework considers the social impact of marketing standards, including how they can lead to food losses and waste and exacerbate food insecurity in certain communities. The framework explores specific ways to address these issues by working with stakeholders to promote equitable access to food and reduce losses and waste in the food supply chain.

The second layer of the framework includes the supply chain stages that need to be thoroughly investigated to identify food losses and waste caused by marketing standards and develop solutions to reduce or eliminate them. These stages are (1) primary production, (2) processing and manufacturing, (3) retail, and (4) consumption. The primary production stage of the supply chain refers to farm or post-harvest level. Food losses in this stage can be caused by the implementation of marketing standards that require farmers to grow specific varieties of crops or to produce food that meets certain aesthetic standards. This can result in farmers discarding crops that do not meet these standards or harvesting crops before they are fully ripe to meet a specific delivery schedule. The framework promotes solutions such as encouraging the use of sustainable agricultural practices that can improve the yield and quality of crops and the implementation of alternative marketing standards. The processing and manufacturing stage of the supply chain involves transforming raw agricultural products into finished products such as packaged goods, canned products, and frozen foods. Food losses in this stage can be caused by the implementation of marketing standards that require a certain appearance or size of raw foods, which can result in the discarding of foods that do not meet these standards. The framework suggests various solutions based on advanced technological solutions and optimized processes. The retail stage of the supply chain involves the distribution and sale of food products to consumers. Food waste in this stage can be caused by marketing standards that require strict policies, such as specific product displays or expiration dates. This can lead to retailers discarding food products that are still edible but do not meet these standards. The framework includes solutions such as improving inventory management systems to reduce overstocking, implementing alternative marketing standards that do not require specific product displays, and adopting sustainable packaging and storage solutions to prolong shelf life and reduce spoilage. Finally, the consumption stage of the supply chain involves the actual consumption of food products by consumers. Food waste in this stage can be caused by consumers purchasing more food than they need or discarding food that has passed its expiration date. The framework focuses on proposing solutions such as improving education and awareness among consumers about the importance of reducing food waste, encouraging the use of meal planning and food preservation techniques, and implementing policies that promote the donation of excess food to food banks and other charitable organizations. Consumption can be shaped in several ways by marketing standards. Higher standards can cause consumers to buy more because they introduce higher-quality products that will make consumers have more confidence in them. However, they may also decrease consumption by leading to raising the price of products and thus limiting the consumer’s access. Moreover, looser standards can also result in cheaper products and more consumption, or on the contrary, lead to less consumption as they may signal that the product is of a lower quality. We identify eight key pathways to capture this dynamic:

1. Looser standards → increased consumption → increased waste: Lower prices encourage greater consumption, but the resulting excess often leads to more waste.
2. Looser standards → increased consumption → decreased waste: Lower prices make imperfect but safe food more accessible, reducing waste by expanding market acceptance.
3. Looser standards → decreased consumption → increased waste: Perceived lower quality diminishes demand, leading to unsold inventory and waste.
4. Looser standards → decreased consumption → decreased waste: Lower demand results in less production, reducing both consumption and waste.
5. Stricter standards → increased consumption → increased waste: Higher quality perception drives overconsumption, leading to more food waste, especially in retail and household stages.
6. Stricter standards → increased consumption → decreased waste: Consumers may value higher-quality products more, resulting in less waste due to better handling or preservation.
7. Stricter standards → decreased consumption → increased waste: Higher prices limit access, causing unsold products to go to waste.
8. Stricter standards → decreased consumption → decreased waste: Reduced demand from high prices may lead to lower production, cutting waste overall.

The linking of these pathways guarantees that the consumption of food is included as an endogenous factor, driving waste throughout the food chain. The framework allows for a more accurate appraisal of how shifting marketing standards, from looser to stricter, impacts consumption and waste so as to provide better focused methods for addressing FLW.

**Figure 2:**
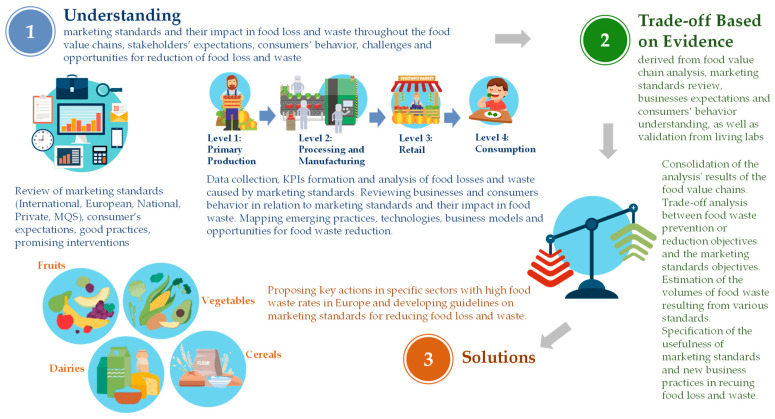
The main implementation activities.

The third and final level of the framework presents the activities that are necessary to be performed in order to effectively tackle food waste and losses related to marketing standards. These activities include (1) marketing standards, food losses and waste research, (2) assessment, and (3) key actions. The first activity of the framework involves conducting research on marketing standards and related food losses and waste in the supply chain. This could involve a comprehensive analysis of current marketing standards and how they impact the supply chain stages, leading to food losses and waste. The research should also identify the different types of food losses and waste, and the reasons for their occurrence, such as overproduction, shelf life issues, and aesthetic standards. The research should not only explore national, EU and international marketing standards but also focus on the reasons for establishing private standards and the potential conflicts they have with minimum quality standards are set by government regulations. The second activity involves assessing the trade-offs between marketing standards and food losses and waste throughout the supply chain stages. It needs to include a trade-off analysis between food losses and waste prevention/reduction objectives and marketing standards objectives. The assessment should also include a cost–benefit analysis of the different marketing standards related to food losses and waste to determine whether the benefits of implementing marketing standards outweigh the costs of implementing and enforcing those standards. In order to perform an effective assessment, it is necessary to also estimate the amounts of food waste that result from applying the different marketing standards. Finally, this activity should lead to presenting suggestions for improving business practices based on the value of food as a driver to reduce food losses and waste. The third activity of the framework concerns conducting specific key actions in four major food categories: fruits, vegetables, dairy products, and cereals, that are analyzed in Section 4 of this paper. These key actions also require thorough testing to evaluate their effectiveness in real-world scenarios. All actions involve collaboration between stakeholders from the different supply chain stages, including farmers, manufacturers, retailers, and consumers. The aim of presenting these actions in detail in Section 4 is to provide insights and prospects on effectively reducing or eliminating food waste and losses throughout the supply chain. This will also lead to developing guidelines based on the research, assessment, and key actions. These guidelines will provide a clear plan for reducing food losses and waste due to marketing standards across the supply chain stages. The guidelines will consider economic, social, and environmental factors, including the impact on farmers’ income, consumer behavior change, and waste reduction targets. To better understand the scope and significance of the third layer of the framework, Figure 2 analyzes its main activities in a nutshell by summarizing their offerings.

The main activities involve the following:

1. Understanding: Performing a comprehensive review and understanding of public and private marketing standards, researching food losses and waste across the different stages of the food chain in order to identify challenges and opportunities for reducing food waste and collect data relevant from stakeholders.
2. Trade-off analysis based on evidence: Assessing the results obtained from the understanding phase, including trade-off analysis between food losses/waste prevention and marketing standards objectives, cost–benefit analysis of different marketing standards, estimation of food losses/waste amounts, and identification of improved business practices.
3. Solutions: A comprehensive set of valuable key actions for reducing and/or eliminating food waste due to marketing standards, offering improved market access while at the same time reducing food poverty, enhanced consumer acceptance, improved technological integration, and enriched sustainable practices. After these actions are tested and evaluated, they will lead to the development of specific guidelines, and best practices will be disseminated.

### 3.2. Analyzing FLW in Four Major Food Sectors and Proposing Relevant Key Actions

The proposed key actions that were mentioned in the framework analysis are carefully designed actions that reflect the diverse characteristics of the food supply chain and include primary production, processing and manufacturing, retail, and consumption stages. The four food sectors included in this key action presentation are fruits, vegetables, dairies, and cereals. Each key action focuses on specific food sectors and involves relevant stakeholders such as producers, processors, retailers, consumers, and waste management organizations. The key actions highlight the social, environmental, technological, and financial impact of marketing standards on food loss and waste. Therefore, they aim to provide different approaches and strategies to reduce food losses and waste due to marketing standards in the supply chain. Each food sector presents distinct consumption and waste dynamics influenced by marketing standards. The following subsections explore how these dynamics unfold within specific sectors, applying the eight pathways to provide nuanced insights into each sector’s unique challenges and opportunities.

#### 3.2.1. Fruits

The fruit supply chain is particularly vulnerable to food waste and losses due to the perishable nature of fruit, which requires careful handling, packaging, and storage to prevent spoilage. Also, fruit waste is compounded by a short shelf life that necessitates quick sales. As a result, fruit that is not handled and stored properly can quickly become unsellable, leading to substantial waste. There are several factors that contribute to food waste in the fruit supply chain. One of the primary reasons is overproduction, where more fruit is grown than is needed or can be sold, leading to excess fruits being thrown away. Inadequate storage and transportation facilities can also lead to spoilage and loss, particularly in regions with poor infrastructure or where transport is difficult. Consumer preferences for visually perfect fruits can also contribute to waste. There are also sustainable packages proposed by marketing standards, such as carton boxes, that need to be improved in terms of effectively protecting the fruits. These packages currently increase the probability of damaging the fruits as they do not offer adequate protection. Therefore, they may be discarded or left unsold, even though they are still edible and nutritious. This can lead to a situation where large quantities of perfectly good fruit are wasted simply because they do not meet aesthetic standards. Addressing food waste in the fruit supply chain is critical for sustainability and for reducing the environmental impact of food production. By working together to improve efficiency, reduce overproduction, and promote sustainable practices, stakeholders can ensure that fruits are produced, distributed, and consumed in a responsible and sustainable manner.

Fruits are more sensitive to particular marketing standards as size, color, and geometric symmetry. Stricter standards (Pathway 5) may lead to a view by the consumers that the quality is superior, thus higher purchasing and utilization. But this also results in overbuying where fruits are spoilt at the household level because they are not sold on time. In addition, big retail stores often discard fruits that are still fresh, but from the physical appearance, do not meet these qualities, which creates a lot of food waste. On the other hand, looser standards (Pathway 2) imply accepting fruits with minimal cosmetic defects and, thereby, raise accessibility and possibly increase per capita consumption rates and reduce waste. This approach is particularly useful where consumers are low-income earners who may not be as keen on the looks of a product as much as its price. The issue here is to find the way to change the consumers’ demand in offering perfectly looking fruits while advancing the sustainability goals and eliminating the fruits’ imperfection rejection by people, thus decreasing the waste rate in the supply chain.

According to the EU Food Loss and Waste Prevention Hub, almost 50% of the food waste generated by households concerns fruits and vegetables [54]. Also, according to a study by the University of Edinburgh, Europe discards over 50 million tons of fruits and vegetables annually due to their failure to meet the expectations of supermarkets and consumers regarding their appearance, with the climate change impact of cultivating the wasted food equaling the carbon emissions of nearly 400,000 cars [55]. Marketing standards can play a significant role in generating food waste in the fruit supply chain. In some cases, marketing standards require that fruit meet certain criteria for size, shape, color, and other visual characteristics in order to be sold. This can result in large amounts of perfectly edible fruit being rejected and wasted simply because they do not meet these criteria. In addition, marketing standards can contribute to overproduction and to the production of fruits that are not well suited to the local climate or soil conditions. To reduce food waste in the fruit supply chain, it is important to develop more sustainable practices that take into account the unique needs and characteristics of each crop and growing region.

Reducing food waste in the fruit supply chain requires collaboration among a range of actors, including producers, processors, distributors, retailers, and consumers. Each of these stakeholders has a role to play in reducing food waste and improving the sustainability of the supply chain. Producers play a critical role in reducing food waste as well as losses in the fruit supply chain by growing and harvesting fruits. Relevant data for producers includes crop yield, harvest timing, and weather conditions. Food processors are responsible for sorting, cleaning, and packing the fruits as well as transforming raw fruit into finished products, such as canned fruit or fruit juice. They can work closely with producers to develop new packaging techniques that ensure the safe transport and storage of fruits. Relevant data for processors includes the amount of fruit received, the amount of waste generated, and the efficiency of processing operations. Distributors are responsible for moving fruit from the processing plant to retailers and other customers. They can collaborate with producers and retailers to create a more sustainable supply chain that reduces waste and greenhouse gas emissions. They can also implement new technologies, such as real-time storage monitoring, to improve the efficiency of transportation and reduce spoilage. Retailers play a critical role in the fruit supply chain by managing inventory levels and monitoring spoilage on store shelves. They can accept to sell fruits that may have imperfections. They can also collaborate with local producers to promote locally grown and seasonal produce. Retailers can also implement zero-waste practices, such as using reusable packaging and reducing single-use plastic. Relevant data for retailers includes sales volumes, inventory levels, and spoilage rates. Consumers are responsible for a significant portion of food waste in the fruit supply chain, as their personal perception of “safe to eat” fruits leads to excessive amounts of fruit waste. They can contribute to fruit waste reduction by choosing to buy fruits that may not meet the cosmetic standards but are still nutritious and delicious. They can also support zero-waste practices by using reusable bags and containers when shopping for fruits. Relevant data for consumers includes purchasing habits, storage practices, and consumption patterns.

**Figure 3:**
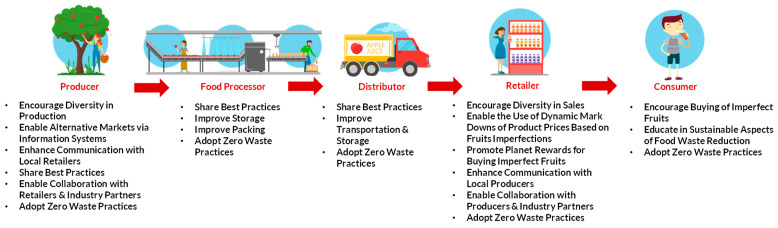
Actions for the fruit sector.

The key to reducing fruit waste is to encourage diversity in production and sales. This would allow for a wider range of fruits to be sold, some of which may be less visually appealing but still nutritious and delicious. Adjustments to marketing standards are necessary so that they become more flexible, such as by accepting fruits that may have slight imperfections. This would reduce the amount of waste produced due to marketing standards. Also, active collaboration between retailers, producers, and industry groups is critical to create a more sustainable food system. This can involve sharing best practices for reducing waste and suggesting new technologies to improve packaging, storage, and transportation, such as smart packaging and real-time storage monitoring of fruits with lower cosmetic standards, as well as analyzing the generated data to improve decision-making. This will ensure that produce is transported quickly and efficiently, reducing the likelihood of spoilage and waste as well as the need for returns and the related greenhouse gas emissions. In this manner, it would be beneficial to implement solutions such as dynamic markdowns of product prices or planet rewards, i.e., financial (tax-deductible) contributions towards NGO-driven projects like Famine Relief (UNICEF) or Reforestation (WWF), to incentivize consumers to buy imperfectly shaped or colored fruits. Also, it is important to achieve a greater connection of local producers with retailers so that locally grown and seasonal produce is promoted, as it reduces the need for long-distance transportation and storage, which can increase the likelihood of spoilage and waste. Moreover, facilitating the process for finding alternative markets using information systems to offer fruits that would be thrown away otherwise is necessary. Finally, promoting zero-waste practices aiming to eliminate waste by reusing, recycling, and composting as much as possible is critical. For example, producers can compost any unsold or imperfect produce to create nutrient-rich soil for future crops, and retailers can use reusable packaging and reduce single-use plastic. A list of all the proposed key actions for the fruit sector throughout the supply chain is presented in Figure 3.

#### 3.2.2. Vegetables

The issue of vegetable waste is a complex and multi-faceted problem that is influenced by several factors. One significant factor contributing to vegetable waste is the strict marketing standards that dictate the size, shape, and aesthetic quality of produce. This can lead to the rejection of vegetables that do not meet these requirements, resulting in significant waste and overproduction. In fact, due to the prevalence of strict marketing standards for decades, consumer expectations have been greatly affected. Consumer preferences for perfectly shaped and unblemished produce can significantly contribute to waste, with many vegetables being discarded due to perceived low quality or aesthetic imperfections. Also, many individuals are overbuying or not using produce before it spoils. The supply chain for vegetables is often long and complex, with several stages where waste can occur. Inadequate storage, transportation, and handling can all contribute to spoilage and waste, particularly in areas where the necessary infrastructure and support are lacking. As a result, vegetable waste may occur during harvest, packaging, shipping, and distribution. Finally, many countries lack the necessary infrastructure and support to effectively monitor vegetable waste, leading to tons of perfectly edible vegetables being thrown away, as well as limited financial and technical resources to develop the necessary solutions. Addressing vegetable waste requires a collaborative effort across all stages of the supply chain, from production to consumption. Strategies to reduce waste may include implementing advanced solutions for packaging and storage, improving transportation and handling, educating consumers on accepting to buy imperfect products and reduce vegetable waste, as well as improving market access. By working together to address these complex and interrelated factors, stakeholders can significantly reduce vegetable waste.

Vegetables are also impacted by strict marketing standards, which often emphasize visual perfection. In the case of stricter standards (Pathway 7), consumer demand may decline if higher prices are a barrier, leading to increased waste at both the retail and household levels as unsold or unpurchased vegetables go to waste. Alternatively, looser standards (Pathway 2) that allow for the sale of vegetables with slight imperfections can promote affordability, thereby encouraging increased consumption and reducing waste. However, there is also the potential for looser standards to reduce consumption if consumers perceive these vegetables as lower quality (Pathway 3), which could lead to increased waste if such produce remains unsold. The key to addressing vegetable waste lies in educating consumers to accept vegetables that may not look perfect but are just as nutritious and safe to eat, thus preventing unnecessary rejection and waste.

According to the 2021 report from the European Commission’s Knowledge Centre for Bioeconomy [56], food waste generated in the EU is approximately 129 Mt along the whole food supply chain, with vegetables (24%) being the group that produces the largest amounts of food waste, followed by fruits (22%). Moreover, it is estimated that 46% of vegetables available at the beginning of the food supply chain become food waste. Marketing standards can have a significant impact on vegetable waste generation. Marketing standards dictate the quality, size, and appearance of vegetables that are sold in the market. These standards may require that produce be of a certain size, color, and shape, with specific levels of defects or blemishes. When marketing standards are too rigid or strict, they can lead to significant amounts of vegetable waste. For example, if a marketing standard requires that all cucumbers sold in the market must be a certain length and width, any cucumbers that do not meet that standard will be discarded as waste, even if they are perfectly good to eat. Similarly, if a marketing standard requires that all tomatoes sold in the market must be a certain shade of red, any tomatoes that are slightly lighter or darker will be discarded. In some cases, marketing standards can also lead to overproduction of certain vegetables, which can result in a surplus that goes unsold and eventually ends up as waste. Farmers may feel pressure to meet these standards and overproduce in order to ensure they have enough produce that meets the standard to sell.

There are several actors involved in the issue of vegetable waste, including producers, food processors, distributors, retailers, consumers, and food banks. Producers are often the first point of contact in the vegetable supply chain. They are responsible for growing and harvesting the produce and can be affected by factors such as weather, pests, and disease. They may also be responsible for overproduction or underproduction of vegetables, which can contribute to waste. Relevant data received from them includes the quantity and quality of produce, as well as the cost of production. Food processors are responsible for processing vegetables into various products such as canned vegetables, frozen vegetables, and vegetable juices. They may also be involved in trimming and packaging produce for retail sale. They can also transform the imperfect produce into value-added products, such as soups or pre-cut vegetable mixes. Relevant data includes the quantity and quality of produce used, as well as the cost and revenue associated with the processing. Distributors are responsible for moving vegetables from processors to retailers and other customers. They are a crucial player in the vegetable supply chain as they facilitate the collaboration between growers to combine their produce into larger quantities that meet the needs of retailers and other buyers. Relevant data includes the amount of produce distributed and the cost and conditions of transportation. Retailers are the ones who sell the vegetables to consumers and can also offer alternative markets for imperfect produce, such as producer’s markets, community-supported agriculture programs, and food cooperatives. Relevant data includes the quantity and quality of produce sold, as well as the revenue and profit associated with the sales. Consumers are the ultimate end users of vegetables and are therefore an important factor in the vegetable waste issue. They may be responsible for overbuying, discarding produce prematurely, or rejecting produce based on aesthetic standards. Relevant data includes consumer preferences, purchase behavior, and feedback. Finally, food banks can play a critical role in reducing vegetable waste. They can receive donations of excess produce that does not meet market standards and distribute it to people in need. Relevant data includes the quantity and quality of produce donated, as well as the cost and efficiency of the distribution process.

**Figure 4:**
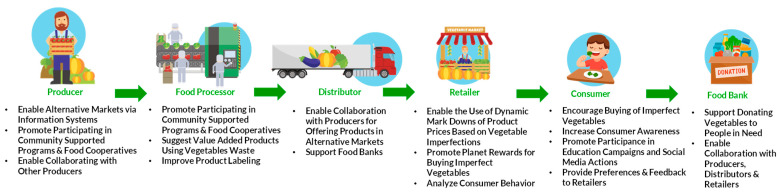
Actions for the vegetables sector.

The key to reducing vegetable waste is ensuring improved market access and consumer acceptance. Actions such as identifying and promoting alternative markets, such as producer’s markets, community-supported agriculture programs, and food cooperatives, that can help growers reach consumers who are interested in purchasing locally produced vegetables that may not meet standard market requirements, are critical. Additionally, it is valuable to create more value-added products, such as soups or pre-cut vegetable mixes, that utilize produce that may not meet standard market requirements but are still nutritious and flavorful. Also, the collaboration between growers to combine their produce into larger quantities that meet the needs of retailers and distributors should be encouraged. Moreover, working with food banks and other charitable organizations to donate excess produce that does not meet market standards to people in need is highly helpful. Also, information systems (platforms and data analytics) can enable producers to find alternative markets for selling the vegetables that would otherwise be wasted. Additionally, increasing consumer awareness about the issue of vegetable waste and the importance of reducing it through education campaigns and social media is useful. Furthermore, implementing dynamic pricing strategies that offer discounts or planet rewards on imperfectly shaped or colored vegetables to encourage consumers to buy them could be critical. Finally, improving product labeling to clearly indicate that vegetables that do not meet market standards are still safe to eat and have the same nutritional value is strongly suggested. Such solutions will lead to personalized approaches for customers with a rich understanding of sensitivity of price and freshness in order to sell vegetables that would otherwise be wasted due to cosmetic standards. A list of all the proposed key actions for the fruit sector throughout the supply chain is presented in Figure 4.

#### 3.2.3. Dairies

Dairy waste can refer to any waste generated during the production of dairy products, such as milk, cheese, and yogurt. This waste can include things like excess milk, whey, and sludge from wastewater treatment systems. Dairy products may pose health risks if they are not consumed on time, and this is why they have labels with expiring dates on them. One major source of dairy waste is excess milk that is not sold or used for processing. This can occur due to overproduction, spoilage, or milk that does not meet the marketing standards required for sale. Cheese and yogurt are two popular dairy products that also contribute to food waste. In the case of cheese, waste can occur during the production process, where milk that does not meet quality standards or excess whey is generated. Additionally, cheese can become moldy or spoiled if not stored properly, leading to waste at the retail and consumer level. Yogurt waste can occur due to excess production or spoilage during storage and transport. Sometimes, yogurt may be subjected to overstocking or remain unsold past its expiry date, causing waste at both the retail and consumer levels. Technology can be a powerful tool in reducing dairy waste. By using advanced techniques and processes, such as sensors and data analytics, new processing techniques, and improved storage and transportation methods, technology can help to reduce the amount of dairy waste generated throughout the production, distribution, and consumption process. Furthermore, technology can also be used to educate and raise awareness among consumers about the issue of dairy waste, helping to encourage more sustainable consumption habits.

In the case of dairy products, stricter marketing standards so as to do with dates of expiry and the condition of the packaging lead to a lot of waste, especially from the retail outlets. For example, fresh stocks of certain products can be rejected to be stocked in the stores due to a short expiration date, even though they are healthy to consume (Pathway 7). In the same respect, the setting of relatively high standards can lead to consumers avoiding purchasing products that are nearly expired, triggering cases of food waste. In contrast, looser marketing standards can lead to more mindful purchasing and clearer labeling, reducing both overconsumption and waste. This could facilitate the opportunity for consumers and retailers to make better decisions as to the safety and lifespan of the products, paving the way to a reduction of waste (Pathway 4). Nevertheless, lower standards (Pathway 1) may further increase consumption, thus, in some instances, contributing to waste production. When dairy products are considered more available and cheaper because of lower standards, consumers buy more than they use, therefore, we see higher rates of spoilage and disposal. Hence, there is a great challenge for balancing flexibility in the marketing standards in order to reduce waste in the flow chain and maintain quality and safety in the dairy products.

Food Navigator Europe [57] has reported that a significant number of dairy products are being wasted, with an estimated 20% of dairy products going to waste. This includes one in six pints of milk being thrown away each year, as well as up to 17% of all yogurts in the EU, which equates to around 6.5 million tons of yogurt being wasted annually. A large proportion of this waste, approximately 55%, occurs within consumers’ households due to spoilage or expiration dates being exceeded. The remaining amount of waste occurs in the dairy supply chain, which includes production, processing, distribution, and sales. Marketing standards play a significant role in generating dairy product food waste. These standards determine the physical characteristics that dairy products should have to be considered marketable, and they can also strongly influence the consumer’s perception of safe-to-eat foods. As a result, dairy products that do not meet these standards are often discarded, even if they are perfectly safe to consume and have the same nutritional value as their counterparts that meet the standards. This leads to a significant number of dairy products being wasted at various stages of the supply chain, from production to retail and consumption. The strict adherence to marketing standards can also lead to farmers overproducing to ensure they meet demand, which can lead to surplus products being discarded due to excess inventory. Additionally, retailers may discard products that are close to their expiration date or have not sold quickly enough, even if the products are still safe to consume. Especially in dairy products, there is a major issue with the improper use of expiration and “best before” dates on labels that needs to be effectively addressed. The former indicates that the product should be consumed before a specific deadline, while the latter suggests that the product can still be safely consumed after that date, provided it has been stored correctly. However, in many EU countries, dairy products only include expiration dates. Shockingly, it has been reported that as much as 80% of yogurt waste in the EU is attributed to products being thrown away when they have passed their expiration date, even though they may still be safe for consumption. Therefore, as it becomes clear, it is important to improve marketing standards across the EU in order to reduce dairy product waste. This situation can be further addressed through increased consumer education on proper food storage and the meaning of date labels, as well as improved communication from food manufacturers and retailers.

Reducing dairy waste requires a collaborative effort from all stakeholders, including farmers, food processors, distributors, retailers, and consumers. The farmers are responsible for producing the dairy products, ensuring their quality, and delivering them to the processors. They can provide data on the quantity and quality of the raw materials used in the production of dairy products, as well as the production processes employed. The processors, on the other hand, are responsible for transforming the raw materials into dairy products, such as milk, cheese, and yogurt. They can provide data on the production process, including the quantity of dairy products produced, the quality of the final products, and the methods used to ensure the freshness and safety of the products. The distributors are responsible for transporting the dairy products from the processing plants to the retailers. They can provide data on the transportation methods used, including the duration of transportation, the temperature of the trucks, and any incidents that occur during transportation. The retailers are responsible for selling the dairy products to consumers. They can provide data on the sales volume, the expiration dates of the products, and any customer feedback on the quality of the products. Finally, the consumers are the end-users of the dairy products. They can provide data on their preferences for dairy products, their purchasing habits, and any feedback on the quality of the products.

**Figure 5:**
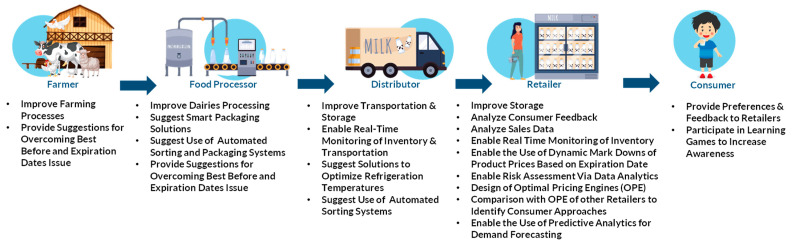
Actions for the dairies sector.

A critical factor for reducing dairy waste is technological innovation. Such innovations include, for example, designing smart packaging that can help monitor the quality of dairy products in real-time, alerting producers and retailers when the product is nearing its expiration date or if there are any issues with the product’s quality. This can help reduce waste by ensuring that products are not thrown away prematurely or sold past their expiration date. Additionally, it would be helpful to implement technological innovations for improving the distribution, and storage of dairy products. For example, cold chain management solutions enable temperature-controlled transportation and storage to ensure that dairy products remain fresh and safe for consumption. Also, automated sorting and packaging systems can help reduce labor costs and improve efficiency in the distribution process, reducing waste due to human error. At the same time, the use of sensors and data analysis can enable optimizing refrigeration temperatures and reduce energy consumption while also ensuring that dairy products remain fresh and safe for consumption. Also, similarly to the dynamic markdowns of product prices or planet rewards based on the cosmetic appearance of vegetables, such dynamic markdowns could be implemented based on the expiration date of the dairy products. More specifically, real-time inventory monitoring of retailers’ dairy SKUs, updating the inventory as soon as an item is sold, as well as monitoring of the expiration dates is strongly suggested to be implemented. Through data analytics, it is essential to perform a risk assessment based on the expiration date of the dairy products and design an optimal pricing engine that will decide the percentage of markdown or planet rewards based on heuristic algorithms. Additionally, the optimal pricing engines of different retailers of the same chain should be compared to improve the accuracy of the engines and understand the customer approaches and their sensitivity of price and freshness. Predictive analytics could also be used to forecast demand for dairy products, allowing producers to adjust their production schedules accordingly. This can help reduce waste by ensuring that products are not overproduced, which can lead to spoilage or the need for markdowns. Furthermore, designing gamification methods to educate consumers about reducing dairy waste could be valuable. By turning the learning process into a game, consumers may be more engaged and motivated to learn and take action. Gamification can also provide a fun and interactive way to teach consumers about the impact of dairy waste on the environment as well as tips and strategies for reducing waste in their own homes. Finally, it would be beneficial to investigate changes in marketing standards, such as overcoming the expiration or “best before” date issue on the dairy product labels, considering the technological advancements that exist nowadays. For instance, smart sensors can track the distribution, storage, and freshness of dairy products and provide real-time updates to consumers, retailers, and distributors, reducing the likelihood of products being discarded prematurely. A list of all the proposed key actions for the dairies sector throughout the supply chain is presented in Figure 5.

#### 3.2.4. Cereals

Food loss in cereals is a complex and multifaceted issue that requires attention from various stakeholders. The production and distribution of cereals are essential for global food security and economic stability, and any loss in the supply chain can have significant consequences. Cereals, such as wheat, rice, maize, and barley, are staple foods that provide a significant proportion of the daily caloric intake for millions of people worldwide. Poor storage conditions, inadequate transportation, and post-harvest losses are the main factors that contribute to food loss in cereals. These factors can cause damage, spoilage, and contamination of the cereals, leading to significant losses. One of the main causes of food loss in cereals is poor storage conditions. Cereals can be damaged by insects, rodents, and pests if they are not stored properly. The storage conditions, such as temperature, humidity, and ventilation, need to be carefully controlled to prevent damage and spoilage. Inadequate transportation is another factor that contributes to food loss in cereals. Cereals can be damaged during transportation due to rough handling, exposure to moisture, and temperature fluctuations. Proper handling and transportation methods, such as using appropriate packaging and containers, can help to reduce food loss. Post-harvest losses also play a significant role in food loss in cereals. Harvested cereals can be lost due to poor handling, processing, and packaging practices. Improper drying and cleaning of grains as well as poor packaging and storage practices, can result in significant losses. The economic impact of food loss in cereals is significant, affecting the income of farmers and other stakeholders in the supply chain. Food loss also reduces the availability of food for consumption, which can lead to hunger and malnutrition, particularly in developing countries. Addressing food loss in cereals requires a coordinated effort among stakeholders, and adopting best practices in storage, transportation, and post-harvest handling can help to reduce food loss in cereals. Technological solutions, such as improved packaging, sorting, and preservation methods, can also aid in reducing food loss. Finally, raising awareness among stakeholders about the specific quality criteria that cereals must meet as well as the importance of reducing food waste can lead to a significant reduction in cereal loss.

Cereal products are viceless to fruits and vegetables when it comes to desirable qualities that are affected, thus leading to waste. Nevertheless, heavy losses can be incurred due to precise normative regulation of moisture content, packaging, and storage. Higher standards that convey quality can lead to increased consumption as consumers may value higher-quality products more, resulting in less waste due to better handling or preservation (Pathway 6). On the other hand, if products do not meet the standards of quality-sensitive customers, then looser standards likely result in more unsold inventory and waste (Pathway 3). Finally, less consumption (Pathway 8) can also be attained via to the implementation of higher standards with an concurrent higher price so that consumers will not be willing to pay more. But this lesser consumption may lead to reduced production and thus to reduced waste as fewer goods are manufactured, which would have otherwise remained unsold in the market. Thus, while stricter standards can create challenges for consumption, they may contribute positively to minimizing waste by ensuring that only high-quality cereal products are available in the market. Maintaining these dynamics is crucial in the realization of improved sustainability within the cereal sector.

Especially in recent years, preserving cereals has become a necessity, with various unexpected circumstances, such as the war in Ukraine, one of the main cereals’ providers, creating significant challenges in the cereals supply chain. According to the 2021 report from the European Commission’s Knowledge Centre for Bioeconomy [56], cereals are in the third place (12%) among the food groups that generate food waste, coming after fruits and vegetables. Marketing standards refer to the rules and regulations that govern the quality and characteristics of food products that are sold in the market. These standards often require cereals to meet specific quality criteria, such as uniform size and shape, specific moisture content, and minimum levels of protein and other nutrients. While these marketing standards can be important for ensuring food safety and quality, they can also contribute to food loss in cereals. For example, if cereals do not meet the specific quality criteria set by marketing standards, they may be rejected by buyers and left unsold. This can result in significant losses for producers and other stakeholders in the supply chain. In some cases, marketing standards may also lead to the rejection of cereals that are perfectly safe and nutritious but do not meet specific appearance or other quality criteria. This can result in significant food loss, as perfectly good cereals are discarded simply because they do not meet certain standards.

There are several actors involved in reducing cereal losses in processing stages, including producers, food processors, and distributors. Producers are responsible for growing and harvesting cereals. They need to be aware of the marketing standards and quality criteria that cereals must meet. They need to provide data on the quality of their crops, including information on crop yield, moisture content, and other quality parameters. Processors are responsible for transforming raw cereals into finished products, such as flour, breakfast cereals, and other food products. They also need to be aware of the marketing standards and quality criteria that cereals must meet, and they need to provide data on the quantity and quality of cereals that they process, including information on processing parameters and final product quality. Finally, distributors play a crucial role in the cereals processing chain as they are responsible for transporting cereals from producers to processors and from processors to retailers. Their role is to ensure that cereals are transported and stored in a way that preserves their quality and prevents food losses. The relevant data that distributors need to provide includes information on the quantity and quality of cereals that they transport and store, as well as information on the transportation and storage conditions. This includes data on the temperature and humidity levels during transportation and storage, as well as information on any incidents or issues that may affect cereal quality, such as spoilage, contamination, or damage.

**Figure 6:**
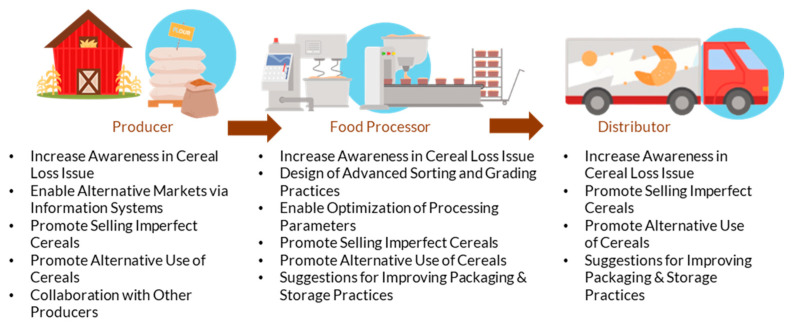
Actions for the cereals sector.

The key actions need to focus on tackling food loss in cereals processing, as this is considered to be the main source of food loss. Firstly, it is necessary to ensure that all stakeholders in the cereals processing chain are aware of the marketing standards and the specific quality criteria that cereals must meet. This can help to reduce the risk of rejection and ensure that cereals are processed and marketed appropriately. Also, developing more flexible marketing standards that take into account variations in crop quality and characteristics can be helpful. This can aid in reducing the risk of rejection and minimize food losses due to quality issues. It would also be beneficial to design advanced sorting and grading practices that can help optimize the processing stages and reduce cereal food losses, such as separating cereals based on their size, shape, color, and other quality criteria through advanced technologies. For example, technologies such as AI and IoT enable identifying and removing cereals that do not meet marketing standards. This can help to reduce the risk of rejection and ensure that cereals are processed and marketed appropriately. At the same time, optimizing processing parameters, such as temperature, moisture content, and processing time, will ensure that cereals meet marketing standards while minimizing food losses. Moreover, supply chain actors should consider developing alternative uses for cereals that are rejected due to marketing standards as well as finding alternative markets via information systems. For example, cereal by-products, such as bran and hulls, can be used for animal feed biofuels or other industrial applications rather than being discarded. This can help to reduce losses and ensure that resources are used more efficiently. In a similar manner, it would be critical to enable and facilitate finding alternative markets using information platforms. Proper packaging and storage practices should also be proposed to reduce food loss in cereal processing. This can involve using advanced packaging materials, such as moisture-proof bags, and ensuring that storage facilities are monitored in real-time. Finally, collaborating with producers to improve production practices can help to ensure that cereals are of appropriate quality and have fewer defects. This can help to reduce losses and ensure that more cereals make it to market. A list of all the proposed key actions for the cereals sector throughout the supply chain is presented in Figure 6.

## 4. Discussion

The proposed FLW framework provides a solid foundation for analyzing FLW along the supply chain, but, with regard to the practical implementation of the concept, it is necessary to discuss further such relevant issues as the nature of the problems that the concept could solve and the possible effects it could bring. The framework has several points of merit, but one of the main ones is the change of marketing standards to permit food that is unphotographable. Nevertheless, the process of translating these changes as applied to various sectors and regions is complex and raises many concerns. For example, the type of consumer motivation that is initiated by aesthetic considerations is cumulative and hence, shifting this type of perception will require time and the implementation of consumer education campaigns, thus the slow return on investment. Furthermore, the implementation of decentralized traceability and reducing waste through technological solutions, such as blockchain and AI, offers attractive opportunities as long as it has some challenges concerning costs, scalability, and the technological support environment, mainly for SMEs and developing countries. Such challenges are important for raising questions related to the costs of implementing such technologies against the desired benefits, including the achievement of waste reduction.

Moreover, the framework provides a holistic approach to FLW management by explicitly integrating the role of food consumption into the supply chain. The eight identified pathways provide a more complex perspective on how marketing standards are related to consumption patterns and the production of waste. By proving these pathways systematically, the framework reveals the inherent trade-offs between promoting consumer confidence through stricter standards and reducing waste by loosening those same standards to accommodate a broader range of products. It also identifies sector-specific forces that shape FLW on this approach. For example, while higher tolerance criteria can facilitate improvement in areas such as fruits and vegetables because more of these commodities will get through the supply line, at the same time it can deter consumer confidence in areas where quality is important, and products have a shelf life, like dairy products. The evidence points to the notion that the assumption of universal marketing standards cannot effectively address FLW. Instead, there is a need to promote specific strategies for each sector, relevant to the behavior of consumers as well as the features of the foods offered. For example, in the fruit and vegetable cases, increasing consumer awareness about ‘ugly’ fruits and vegetables to make these socially acceptable for purchase is one of the most effective ways to reduce waste. Similarly, in the dairy case, improvement in packaging material and date labeling may be the most effective way to prevent waste.

However, while the research attempts to address numerous challenges related to food loss and waste due to marketing standards, several limitations exist within the scope of the research. Firstly, despite comprehensive data collection efforts, the sheer complexity of the food supply chain and the variability in marketing standards across different regions pose challenges in achieving a fully representative sample. Additionally, the research’s focus on specific food sectors may result in a limited generalizability of findings to other sectors within the food industry. Finally, the dynamic nature of marketing standards and evolving consumer preferences may render some research findings outdated over time. Also, further light has to be shed upon the universality of the presented framework. It has to be understood that the context of the research is limited as the actual food supply chain is functioning in very different legal, economic, and social conditions. For example, in some parts of the world, marketing standards may not be a major source of FLW due to other constraints, such as weak infrastructure, limited storage, and transportation facilities. In such situations, asking consumers or developing new technologies could do the trick without having to address some of the structural issues. Thus, it appears that more research should be conducted to build regional variations of the framework that would respond to local circumstances. The framework’s impact can be profound for policymakers and stakeholders within the established industries. The framework could be used by policymakers to alter current rules on marking standards, specifically from the aspect of accommodation of flexibility concerning the physical appearance of the food products. Such policy changes could not only save waste but also bring new markets for ‘defective’ foods, benefiting producers and minimizing hunger levels. From the view of business for industry practitioners, applying the framework could benefit both sustainable businesses as well as increasing potential profitability.

Identified gaps in the research primarily revolve around the need for further exploration into the nuanced interactions between marketing standards and food loss/waste across the supply chain. Specifically, there is a gap in understanding how variations in marketing standards impact different stakeholders, including small-scale producers and marginalized communities. Additionally, while the research emphasizes environmental, economic, and social sustainability, there remains a gap in quantifying the long-term socio-economic impacts of proposed interventions. Moreover, the framework’s focus on European contexts may overlook insights from global perspectives, limiting the applicability of findings in diverse cultural and geographical settings.

Finally, future research efforts should aim to address these limitations and gaps by adopting a more focused approach to understanding the complexities of food loss and waste due to marketing standards. This entails exploring innovative methodologies, such as machine learning algorithms and blockchain technology, to enhance data collection and analysis processes. Furthermore, future studies should strive to incorporate the voices of diverse stakeholders, including policymakers, consumer advocacy groups, and civil society organizations, to ensure inclusive decision-making processes. Additionally, expanding research efforts to encompass global perspectives and emerging markets can provide valuable insights into addressing food loss and waste on a broader scale. Ultimately, future research goals should focus on fostering interdisciplinary collaborations and leveraging emerging technologies to develop sustainable solutions that promote efficiency, equity, and resilience across the food supply chain.

## 5. Conclusions

This paper presents a comprehensive framework aimed at addressing FLW caused by marketing standards across the entire food supply chain, with a particular focus on four key sectors, which include fruits and vegetables, dairy products, and cereals. The research holds significant importance and contributes significantly to the research community tackling food loss and waste due to marketing standards. By addressing a multifaceted issue that intersects environmental, economic, and social dimensions, this research offers holistic insights into the complexities of food supply chains. Through its comprehensive framework, which integrates sustainability pillars, supply chain stages, and other activities, the research provides a structured approach to understanding, mitigating, and preventing food loss and waste. This research not only sheds light on the detrimental impacts of stringent marketing standards but also proposes specific solutions to tackle food waste in four major food sectors. By engaging stakeholders across the supply chain and leveraging key actions, the research extends beyond academic discourse, fostering tangible advancements in sustainable food production, consumption, and waste management practices on both local and EU scales.

Moreover, this research demonstrates that it is possible to significantly reduce food waste due to marketing standards if considerable changes are made in the assessment of consumer preference and the application of technology. The structure of the work presents a clear prospect to extend the findings further to different global settings and also to compare the long-term socio-economic and environmental impact of the proposed solutions. It is crucial to address those aspects to foster improvement of the food system and reduce waste while improving the economy and the general welfare of man. Through urging a reconsideration of marketing standards, particularly the aesthetic ones, and promoting the use of advanced technologies like AI and blockchain for improved traceability, the research aims to close the gap between the sustainable agenda and business priorities. It also becomes clear that marketing standards have an impact on FLW not only by shaping the quality and availability of food products but also by affecting consumption behavior. Nevertheless, the effects of such standards are very sensitive to sectors of origin, hence the call for a differentiated approach for each food sector.

In conclusion, the research created a theoretical guide for limiting FLW originating from marketing standards; however, its broad application requires additional empirical confirmation. For instance, research studies might evaluate the economic and social costs of implementing modified marketing conventions and evaluate the extended value of leveraging technology such as artificial intelligence and blockchain within supply networks. Further research may seek to know how consumer behavior modifies in relation to the number of educational campaigns and policy measures initiated. Finally, further extension of the framework to other regions and sectors will be instrumental for managing the global problem of food waste and reaching the UN Sustainable Development Goals.

## Data Availability

The original contributions presented in the study are included in the article, further inquiries can be directed to the corresponding author.
